# Sequential Activation of Lateral Hypothalamic Neuronal Populations during Feeding and Their Assembly by Gamma Oscillations

**DOI:** 10.1523/JNEUROSCI.0518-24.2024

**Published:** 2024-09-10

**Authors:** Mahsa Altafi, Changwan Chen, Tatiana Korotkova, Alexey Ponomarenko

**Affiliations:** ^1^Institute of Physiology and Pathophysiology, Friedrich-Alexander-Universität Erlangen-Nürnberg (FAU), Erlangen 91054, Germany; ^2^Institute for Systems Physiology, Faculty of Medicine, University of Cologne/University Clinic Cologne, Cologne 50931, Germany; ^3^Max Planck Institute for Metabolism Research, Cologne 50931, Germany; ^4^Excellence Cluster on Cellular Stress Responses in Aging Associated Diseases (CECAD) and Center for Molecular Medicine Cologne (CMMC), Cologne 50931, Germany

**Keywords:** cell assemblies, electrophysiological recordings, feeding, graph-based clustering, innate behaviors, machine learning

## Abstract

Neural circuits supporting innate behaviors, such as feeding, exploration, and social interaction, intermingle in the lateral hypothalamus (LH). Although previous studies have shown that individual LH neurons change their firing relative to the baseline during one or more behaviors, the firing rate dynamics of LH populations within behavioral episodes and the coordination of behavior-related LH populations remain largely unknown. Here, using unsupervised graph-based clustering of LH neurons firing rate dynamics in freely behaving male mice, we identified distinct populations of cells whose activity corresponds to feeding, specific times during feeding bouts, or other innate behaviors—social interaction and novel object exploration. Feeding-related cells fired together with a higher probability during slow and fast gamma oscillations (30–60 and 60–90 Hz) than during nonrhythmic epochs. In contrast, the cofiring of neurons signaling other behaviors than feeding was overall similar between slow gamma and nonrhythmic epochs but increased during fast gamma oscillations. These results reveal a neural organization of ethological hierarchies in the LH and point to behavior-specific motivational systems, the dysfunction of which may contribute to mental disorders.

## Significance Statement

The lateral hypothalamus (LH) is pivotal for regulation of innate behaviors, yet the organization of LH neuronal populations at fine temporal resolution within behavioral episodes remains unknown. Such knowledge would be crucial for understanding the contribution of LH to different innate behaviors. Here, we identified distinct groups of LH cells active at specific times within feeding bouts and, additionally, populations more continuously active during feeding, exploration, or social interaction. Cells from feeding-related populations coalesce during LH slow gamma oscillations, while fast gamma also promotes assembly across multiple behavioral populations. Our findings suggest that appetitive behaviors and phases of consummatory acts are supported by distinct LH populations. Dysfunction of their interaction and plasticity during network oscillations may contribute to eating disorders.

## Introduction

In humans and other mammals, innate behaviors maintain physiological homeostasis, organize interactions with conspecifics to ensure safety and reproduction, and facilitate learning through exploratory responses to novelty. Innate behaviors are regulated by the hypothalamus, which processes various chemical, sensory, and cognitive control signals (Tinbergen, 1951; Swanson, 2000). Hypothalamic output to forebrain and midbrain regions is mainly conveyed by the anatomically complex and functionally diverse lateral hypothalamus (LH), crucial for the coordination of multiple innate behaviors (Bonnavion et al., 2016; Herrera et al., 2017; Stuber, 2023; C. Chen et al., 2024). Seminal studies showed that LH lesions dramatically reduce food intake and lead to starvation (Anand and Brobeck, 1951; Grossman et al., 1978), while the LH stimulation elicits feeding (Hoebel and Teitelbaum, 1962; Margules and Olds, 1962). These effects are brought about by functionally and neurochemically distinct LH neurons which receive appetitive and anorectic signals from within the hypothalamus, basal forebrain, and hindbrain regions (Jennings et al., 2013, 2015; Nieh et al., 2015; O'Connor et al., 2015; Carus-Cadavieco et al., 2017), respond to glucose (Oomura et al., 1974), and project to various regions within the feeding circuitry (Sternson and Eiselt, 2017). Separate populations of LH neurons are involved in food intake and reward, depending on their molecular identity and projections (Marino et al., 2020; Siemian et al., 2021b). Circuits mediating interactions with conspecifics also involve LH, where cells with partially different molecular identities are excited or inhibited during social behaviors (Nieh et al., 2016; Bai et al., 2023; Petzold et al., 2023; C. Chen et al., 2024). Interactions within and between these neuronal populations allow behaviors to be prioritized according to homeostatic needs and sensory cues (Petzold et al., 2023; Barbano et al., 2024). Studies of the other major output of the hypothalamus, the paraventricular nucleus, have highlighted the possibility that different subpopulations of cells with overlapping molecular profiles may be involved in disparate phases of food intake including its initiation, increasing satiation and meal termination (C. Li et al., 2019; M. M. Li et al., 2019). Orosensory and gastrointestinal distention signals are processed by different populations of a key hypothalamic afferent region in the brainstem, generating sequential negative feedback signals during food intake (Ly et al., 2023). It is currently unknown whether the hypothalamus features feeding phase-specific cell populations and, of particular importance for LH, whether they are also involved in other innate behaviors.

Network oscillations influence the timing of neuronal discharge and coordinate signaling within and across brain regions (Gray et al., 1989; Buzsáki and Watson, 2012) including the hypothalamus (Carus-Cadavieco et al., 2017; Samerphob et al., 2019; Tsurugizawa et al., 2019; C. Chen et al., 2024). Gamma oscillations have been implicated in feeding behavior. Food seeking in sated mice involves coordinated gamma rhythmic activity in the LH, lateral septum, and prefrontal cortex (Carus-Cadavieco et al., 2017) and is characterized by increased LH–ventral striatum gamma band coherence upon repeated contact with palatable food (Samerphob et al., 2019). Gamma local field potential (LFP) oscillations at the location of primary energy sensor neurons in the ventromedial hypothalamus were reduced in energy-deficit states in fasted mice (Tsurugizawa et al., 2019) and in mice with weight loss induced by anorexia during chronic treatment with the chemotherapeutic cisplatin (Sun et al., 2021). The impact of gamma oscillations on neuronal activity in the hypothalamus has been revealed at the resolution of single oscillation cycles (Carus-Cadavieco et al., 2017), where populations of cells preferentially fire at certain oscillation phases, thereby jointly determining efferent signaling (O'Keefe and Recce, 1993; Dejean et al., 2016). On the other hand, network synchronization during gamma oscillations involves increased activity in the population of participating neurons (Csicsvari et al., 2003; Ainsworth et al., 2012; Carus-Cadavieco et al., 2017; Sherfey et al., 2018). The joint participation of neurons in oscillations, indicated by the degree of their correlated discharge, supports their direct and polysynaptic effects on each other and on their efferent cells and influences synaptic plasticity (Csicsvari et al., 2003; Kim et al., 2016; Galuske et al., 2019). However, the organization of LH cell assemblies according to their behavioral specificity during gamma oscillations, potentially supporting the coordination of functionally similar or diverse neurons, remains unknown.

Here we used spectral clustering to analyze firing rate dynamics of LH neurons during different behaviors. Graph-based spectral clustering, which outperforms traditional methods such as *k*-means and hierarchical clustering (Namboodiri et al., 2019; Kariotis et al., 2021), is particularly useful for classifications of nonlinear dynamics of functionally heterogeneous neural populations (Gouwens et al., 2019; Hirokawa et al., 2019; Namboodiri et al., 2019). We found that separate populations of LH neurons recorded in a free choice model are active at particular times during feeding episodes. Other populations of LH cells were more active during social interaction and exploration of novel objects than during feeding. LH gamma oscillations were associated with either increased or decreased cofiring of neurons as compared with nonrhythmic epochs. LH cells from different groups showed distinct assembly patterns during slow and fast gamma oscillations suggesting that distinct types of neuronal synchronization in the LH specifically support feeding and facilitate the coordination of behavior-specific cells.

## Material and Methods

### Experimental procedures

Experimental data in this study have been used in C. Chen et al. (2024) where data collection is described in detail. Briefly, 10- to 25-week-old Vgat-ires-Cre knock-in mice (The Jackson Laboratory, used also in optogenetic stimulation experiments after recordings of spontaneous behaviors) and C57BL/6 male mice were housed under standard conditions with a 12 h light/dark cycle. The firing rate during behaviors of cells recorded in three C57BL/6 and one Vgat-ires-Cre mice (The Jackson Laboratory) was compared to ensure the absence of electrophysiological differences between the mouse lines. Prior to the experiments, mice were handled and habituated to the experimental enclosure for 3–5 d. Four-shank silicon probes (B32, NeuroNexus Technologies) were mounted on custom-made microdrives and stereotactically implanted in the LH at the following coordinates: AP, −1.58; ML, 0.8; medial shank; and DV, 5 mm. Reference and ground electrodes were screws above the cerebellum. The implant was shielded with copper mesh and secured to the skull with dental acrylic.

### Data acquisition and preprocessing

The recording setup consisted of a custom enclosure with two interconnected compartments (LWH 25 × 30 × 20 cm each), with access to water, food, a conspecific, and a novel object (C. Chen et al., 2024). Mice moved *ad libitum* within the enclosure during recordings. Silicon probes were connected to a Neuralynx preamplifier to reduce cable movement artifacts. Signals were amplified, bandpass filtered (1 Hz–8 kHz), and acquired continuously at 32 kHz using Digital Lynx system (Neuralynx). Behavioral recordings were performed by four cameras capturing movement from different angles at 25 Hz (Motif, Loopbio). Ethograms were created using Adobe Premiere and frame-by-frame analysis of synchronized multiangle videos. Feeding was scored when the resident mouse consumed (i.e., ate) food pellets, with the minimal duration of scored feeding bouts of 4.8 s. Sniffing or tracking an intruder mouse was scored as social interaction. Novel object exploration included behaviors such as sniffing, gnawing, touching, or climbing a novel object.

Electrophysiological signals were preprocessed using the Neurophysiological Data Manager (NDManager, http://neurosuite.sourceforge.net/) and analyzed using custom-written MATLAB scripts (MathWorks). In a high-pass–filtered signal, action potentials (spikes) were identified, and spike waveforms were represented by the first three principal components and action potential amplitudes. Automatic spike sorting (https://github.com/klusta-team/klustakwik) was followed by manual cluster adjustments using auto- and cross-correlations of spike trains, Mahalanobis isolation distance between clusters (64 ± 2 across four mice), and visual comparison of waveform profiles across channels (see also Extended Data Fig. 1b in C. Chen et al., 2024). The data were obtained from an average of 82 recordings by individual probe shanks per mouse, i.e., separate spike sorting sessions, resulting in 5 ± 1 sorted units per shank/recording.

### Firing rate dynamics

For individual cells, the firing rate was estimated by the convolution of spike trains with a Gaussian kernel of the size of 500 ms. To obtain the firing rate dynamics vectors for each cell during each of the innate behaviors in a temporally aligned fashion, we standardized episodes of different durations to the average duration of episodes in a recording session (Hirokawa et al., 2019). Specifically, the average duration of behavioral episodes of a given behavior in a recording session was computed, and the number of bins corresponding to the 10 ms resolution in the average episode was estimated. All behavioral episodes in a session were then partitioned using the estimated number of bins. The average firing rate for each bin was computed across different episodes of a given behavior. The firing rate vectors corresponding to three distinct behaviors—feeding, social interaction, and novel object exploration (13,914, 1,774 and 2,039 bins, respectively)—were concatenated for each cell and normalized using *z*-score transformation. To mitigate the impact of the behaviors duration variability and yield robust clustering (Jolliffe, 2002), we further projected the *z*-scored firing rate vectors onto a lower-dimensional space through principal component analysis prior to clustering. The first 128 principal components were retained, capturing 90% of the variance of the firing rate vectors.

### Spectral clustering

Clustering analysis was performed on the 128-dimensional firing rate vectors of LH cells using the Scikit-learn package. Spectral clustering identifies data partitions with fewer intergroup connections and denser intragroup links and is particularly stable in higher-dimensional datasets (Von Luxburg, 2007; Hirokawa et al., 2019). The affinity matrix for clustering was computed using a *k*-nearest neighbor (*k*-NN) connectivity matrix and the cosine metric as the similarity measure for the firing rate vectors. To determine the optimal combination of the number of clusters and *k*-NN, which influence graph construction and thus the clustering output, we used stability analysis through bootstrapping without replacement 100 times, randomly selecting 90% of the data for each iteration (Hirokawa et al., 2019). To evaluate the stability of clustering for various combinations of cluster numbers and *k*-NN, we computed the adjusted Rand index (ARI; Steinley, 2004) and adjusted mutual information (AMI) score (Vinh et al., 2010). These independent metrics assess clustering stability across subsamplings as the proportion of cells with consistent clustering and the mutual dependence between the clusterings, respectively. Optimal clustering parameters (number of clusters and *k*-NN) were then determined as the maximal mean and mean adjusted for variance (mean/standard deviation ratio) of ARI and AMI across bootstrapped distributions. Importantly, the selected parameters enabled reliable clustering as opposed to random assignment of cells to clusters, as indicated by both the ARI and AMI score being well above zero (Steinley, 2004; Vinh et al., 2010).

While stability analysis evaluates the robustness of clustering, it does not reveal its quality, i.e., the similarity of data within clusters and their dissimilarity between clusters. For instance, low intracluster correlations may be an indication of subclusters within a cluster and the need for further segmentation. Clustering quality was estimated by Pearson’s correlations between the first five principal components of firing rate vectors for cells from the same or different clusters.

To further ensure robust clustering quality, we calculated silhouette scores (Rousseeuw, 1987), which measure the similarity of individual data samples to their assigned clusters compared with other clusters using intracluster and nearest-cluster distances, across 100 clustering iterations. Neurons consistently assigned to their respective clusters, i.e., with a silhouette score above 0 for at least 95 of the iterations, were considered for further analysis.

### LFP analysis

The LFP was obtained by downsampling of the wide-band signal to 1,250 Hz using the NDManager. Slow and fast gamma oscillations were detected in the 30–60 and 61–90 Hz bandpass filtered, rectified, and smoothed signal (Carus-Cadavieco et al., 2017). Events with the amplitudes exceeding 2 SD above the noise mean for at least 25 ms were detected. The beginning and the end of oscillatory epochs were designated at times when the amplitude fell below 1 SD (Csicsvari et al., 2003). To obtain the times of nonrhythmic epochs, delta (2–4 Hz), theta (5–10 Hz), beta (15–30 Hz), and gamma (up to 120 Hz) as well as faster oscillations in the 120–200 Hz band were detected (Bender et al., 2015; Carus-Cadavieco et al., 2017; C. Chen et al., 2024). Epochs without detected oscillations exceeding 0.5 SD above the noise mean were marked as nonrhythmic. The multitaper method (NW, 3; window length of 1,024) was used to compute power spectral density.

### Cross-correlations

Cross-correlograms (CCG) of cells recorded by different shanks of a silicon probe were computed using spikes during gamma or nonrhythmic epochs as trigger events and normalized by the cumulative CCG spike count. Cofiring probability was computed as the mean firing probability within ±15 ms of the trigger event. The cofiring ratio (CFR) was calculated for each cell pair as the ratio of the cofiring probability during nonrhythmic epochs to that during gamma oscillations (Fig. 6*c*). To robustly compare the cofiring of LH cells during gamma oscillations versus nonrhythmic epochs, we computed the CCG of the same set of cells after shifting the trigger spike time by a random variable from a uniform distribution in the [−25 25] s interval. This procedure was repeated to obtain 1,000 shuffled CCGs and control CFRs for each cell pair.

### Statistical analysis

Python (https://www.python.org/) and MATLAB (MathWorks) were used for statistical analyses. Two-tailed *t* tests and Mann–Whitney or Kolmogorov–Smirnov (KS) tests were used for individual two-group comparisons, depending on the normality of the distributions. ANOVA, Tukey–Kramer post hoc tests, or multiple two-group tests with Bonferroni’s *α* correction were used for multiple group comparisons. Descriptive statistics are presented as mean ± SEM unless otherwise indicated.

### Data availability

Source data for Figures 1–8 and LH spike trains are available via Figshare (https://doi.org/10.6084/m9.figshare.22317091; source data via https://doi.org/10.6084/m9.figshare.26539723). Further datasets supporting the findings of the current study are available from corresponding authors upon reasonable request.

### Code availability

Code used in the current study is available via GitHub (https://github.com/NeuroAnalyz/Graph-based_clustering) or from the corresponding authors upon reasonable request.

## Results

### Unsupervised classification of LH cells firing rate dynamics

**Figure 1.**
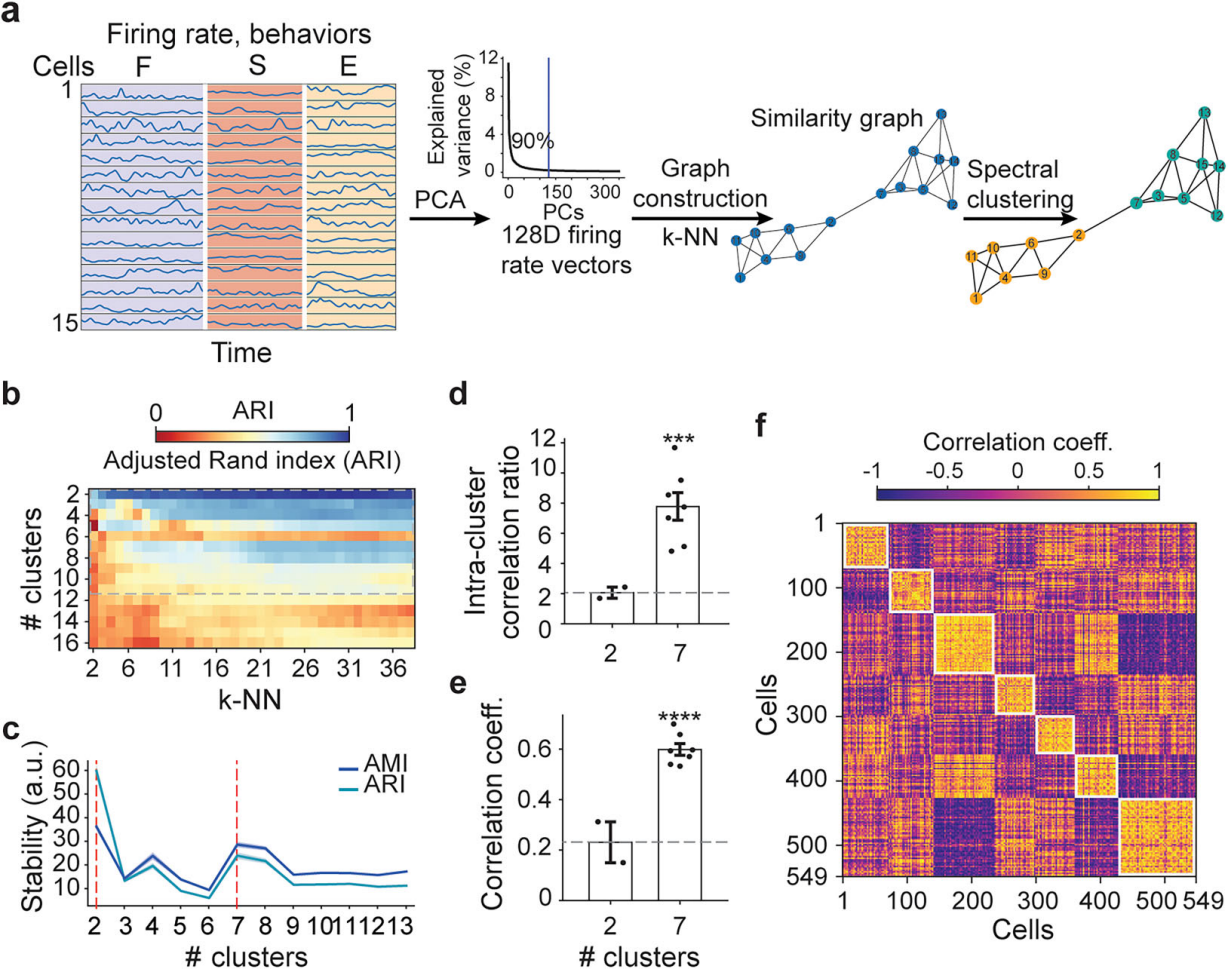
Clusters of LH cells revealed by the spectral clustering of firing rate dynamics during innate behaviors. ***a***, Scheme of the spectral clustering of firing rate vectors of 15 representative neurons during feeding (F), social interaction (S), and exploration (E). PCs, principal components. ***b***, ***c***, Clustering bootstrap stability analysis. ***b***, ARI, mean of 100 bootstrap sessions, for different numbers of clusters and *k*-NN (*n* = 917 cells from 4 mice). Dashed frame, Number of cluster range with high clustering stability, estimated in ***c***. ***c***, Variance-adjusted ARI and AMI (mean across bootstraps/SD) for different number of clusters, mean ± SEM across *k*-NN from 2 to 38. Red dashed lines, Two most stable clusterizations. ***d***, Intra- to intercluster Pearson’s correlation ratio for two- versus seven-cluster analysis. One-sample *t* test, seven clusters versus the mean of two clusters (dashed line); *t*_(6)_ = 6.24; ***p = 0.0007. ***e***, Intracluster correlations for two- versus seven-cluster analysis. One-sample *t* test, seven clusters versus the mean of two clusters (dashed line), *t*_(6)_ = 15.91; ****p < 0.0001. ***f***, Correlations of firing rate feature vectors (first 5 PCs) of 549 cells grouped into seven clusters show high intracluster correlations (squares near the diagonal) and low intercluster correlations.

The firing of 1,349 LH cells was recorded in four male mice using movable silicon probes in a free choice model during eating standard chow, interacting with a younger mouse of the same sex, or exploring a novel object (hereafter, exploration). These cells were recorded in parallel with at least one more cell at another silicon probe shank (see joint firing of LH cells during gamma oscillations and nonrhythmic epochs). Individual LH cells showed variable firing rate dynamics during the three behaviors. We examined the high-dimensional firing rate vectors using an unsupervised approach, spectral clustering, which identifies communities of cells connected by a similarity graph (illustrated for an example subset of cells in Fig. 1*a*). To estimate the optimal number of clusters and *k*-NN, stability analysis was performed using ARI for different combinations of parameters yielding high stability scores for the number of clusters between 2 and 11 with *k*-NN >20 (Fig. 1*b*). Further assessment of the AMI and ARI distributions indicated the most stable clustering using two followed by seven number of clusters (Fig. 1*c*). To compare the clustering quality between two- and seven-cluster approaches (using *k*-NN = 21), we computed the intracluster correlation ratio, which indicates the correlation of the data within clusters compared with correlation of the data between clusters. The dataset segmented into seven clusters showed higher intracluster correlations than with the two-cluster approach, also in relation to intercluster correlations (intracluster correlation ratio two vs seven clusters; *t*_6 _= 6.24; *p* = 0.0007; intracluster correlation two vs seven clusters; *t*_(6)_ = 15.91; *p* < 0.0001; one-sample *t* tests; Fig. 1*d*–*f*). Thus, segmentation of the data into seven clusters was highly reproducible and properly represented the similarity of firing rate dynamics between LH neurons.

### Distinct LH populations signal feeding phases and further innate behaviors

**Figure 2.**
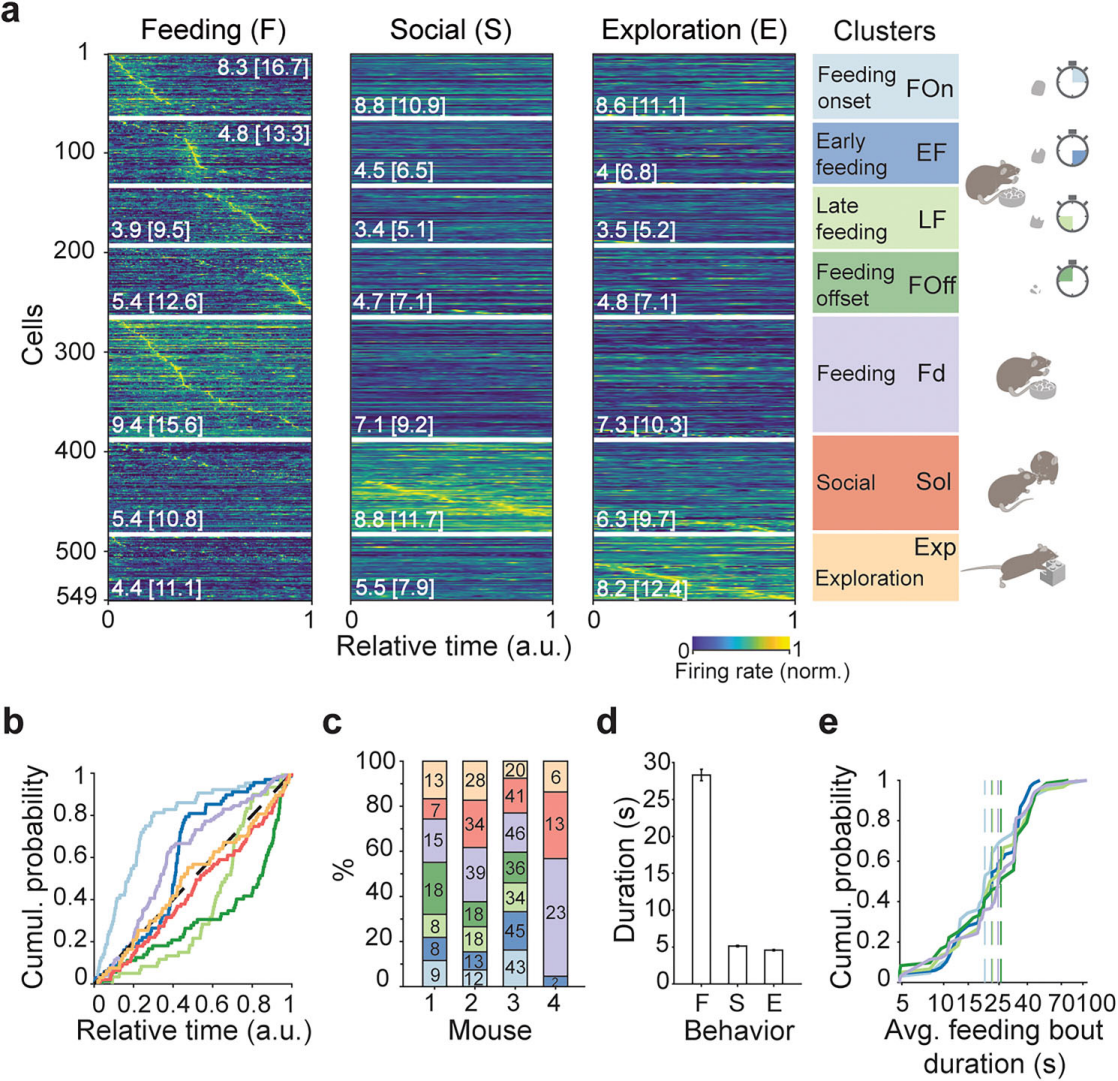
Decoding of feeding phases and innate behaviors by LH cells's firing rate. ***a***, Normalized firing rate dynamics of cells (rows) in each cluster (with margins indicated by white horizontal lines) during feeding, social interaction, and novel object exploration (columns). Data for individual cells are sorted according to the relative time of the maximal discharge in the concatenated firing rate vectors of the three behaviors. Numbers indicate average and, in brackets, peak firing rate (Hz) of cells during each behavior (mean across cells in a cluster). Clusters labeled based on the behavior when the average peak firing rate of cells was the highest: FOn, *n* = 64 cells from three mice; EF, *n* = 68 cells from four mice; LF, *n* = 60 cells from three mice; FOff, *n* = 72 cells from three mice; Fd, *n* = 123 cells from four mice; Sol, *n* = 95 cells from four mice; Exp, *n* = 67 cells from four mice. The color code of neuronal populations is maintained throughout the figure. Note that, unlike FOn, EF, LF, and FOff populations that are active at specific phases of feeding bouts, the instants of maximal discharge in homogeneously active Fd, Sol, and Exp reflect much less pronounced elevations of the firing rate (Fig. 4). ***b***, Empirical cumulative distribution of cells’ peak firing times in the functional populations during behaviors of their maximal discharge, i.e., during feeding, exploration, and social interaction. One-sample KS test for the standard uniform distribution (dashed line), adjusted *α* = 0.0023; FOn, *D* = 0.51; EF, *D* = 0.33; LF, *D* = 0.33; FOff, *D* = 0.38; Fd, *D* = 0.26; *p* < 0.0001 for all feeding-related populations; Sol, *D* = 0.11; *p* = 0.22; Exp, *D* = 0.08; *p* = 0.78. ***c***, Functional LH populations in different mice. Numbers in bars, *n* of cells. ***d***, Average duration of behaviors during which cells were recorded, mean ± SEM across *n* = 549 cells from four mice. ***e***, Empirical cumulative distribution of feeding bouts duration in recordings of cells from feeding-related clusters. Median of distributions, vertical dotted lines, FOn, 19.7 s; EF, 22.2 s; LF, 22.3 s; FOff, 25.7 s; Fd, 24.6 s; Mann–Whitney test; adjusted *α* = 0.005; not different between clusters; *p* > 0.13. *X* axis, log scale.

To functionally characterize LH cells clustered on the basis of similar firing rate dynamics, we investigated their activity in relation to innate behaviors. We examined their firing rate patterns during feeding, exploration, and social interaction. Remarkably, even though behavior was not a predefined label in our unsupervised classification, cells in different clusters (henceforth, populations) fired closely matching these behaviors (Fig. 2*a*). The cumulative probability distributions of the time at which cells fired at their highest rate during their preferred behavior (i.e., the one with the highest peak firing rate across the three behaviors) were not uniform for cells from five populations active during a feeding bout (Fig. 2*b*; one-sample KS test; adjusted *α* = 0.0023; *p* < 0.0001). Four of these feeding-related populations clearly corresponded to temporally different phases of a feeding bout (Fig. 2*a*,*b*). These distinct feeding phase populations were sequentially active from the feeding onset (FOn), through early and late feeding (EF and LF), to feeding offset (FOff). Cells from the exploration and social interaction populations (Exp and Sol) fired homogeneously during these respective behaviors (one-sample KS test; adjusted *α* = 0.0023; exploration, *p* = 0.78; social, *p* = 0.22) and, together with a similar, fifth, feeding population (Fd), changed their average firing rate as a function of behavior (two-way ANOVA; population × behavior; *F*_(4,846) _= 2.01; *p* = 0.09; population, *F*_(2,846) _= 1.37; *p* = 0.25; behavior, *F*_(2,846) _= 0.30; *p* = 0.74). Importantly, the populations were not only evident in the combined data from multiple mice but also within individual animals (Fig. 2*c*). Moreover, within each animal, the proportion of cells in each identified population was similar except for one animal with a lower number of recorded neurons (Fig. 2*c*).

**Figure 3.**
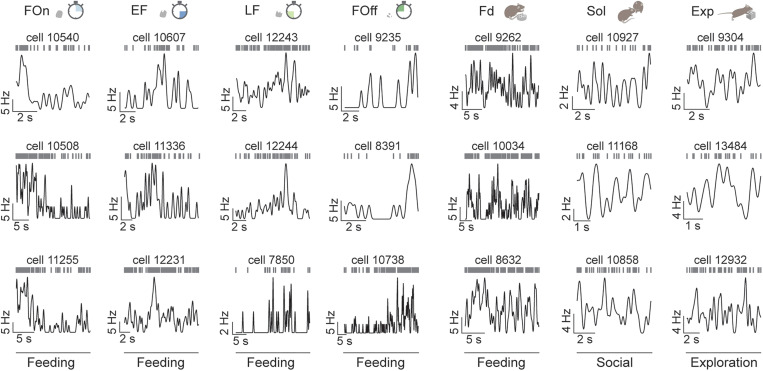
Firing patterns of example LH cells from functional populations during episodes of innate behaviors. Columns, three example cells from each population. Each panel includes the following: top, raster plot of spike train; bottom, instantaneous firing rate over the duration of the behavior.

Feeding bouts were on average six times longer than exploration or social interaction episodes (Fig. 2*d*). Therefore, different feeding phase populations could be activated with progressively longer feeding or, alternatively, signal phases of feeding bouts, regardless of their duration. The duration of feeding episodes was consistent across different feeding-related populations (Mann–Whitney test; adjusted *α* = 0.005; *p* > 0.13; Fig. 2*e*). Thus, those LH populations signal phases rather than the duration of feeding.

**Figure 4.**
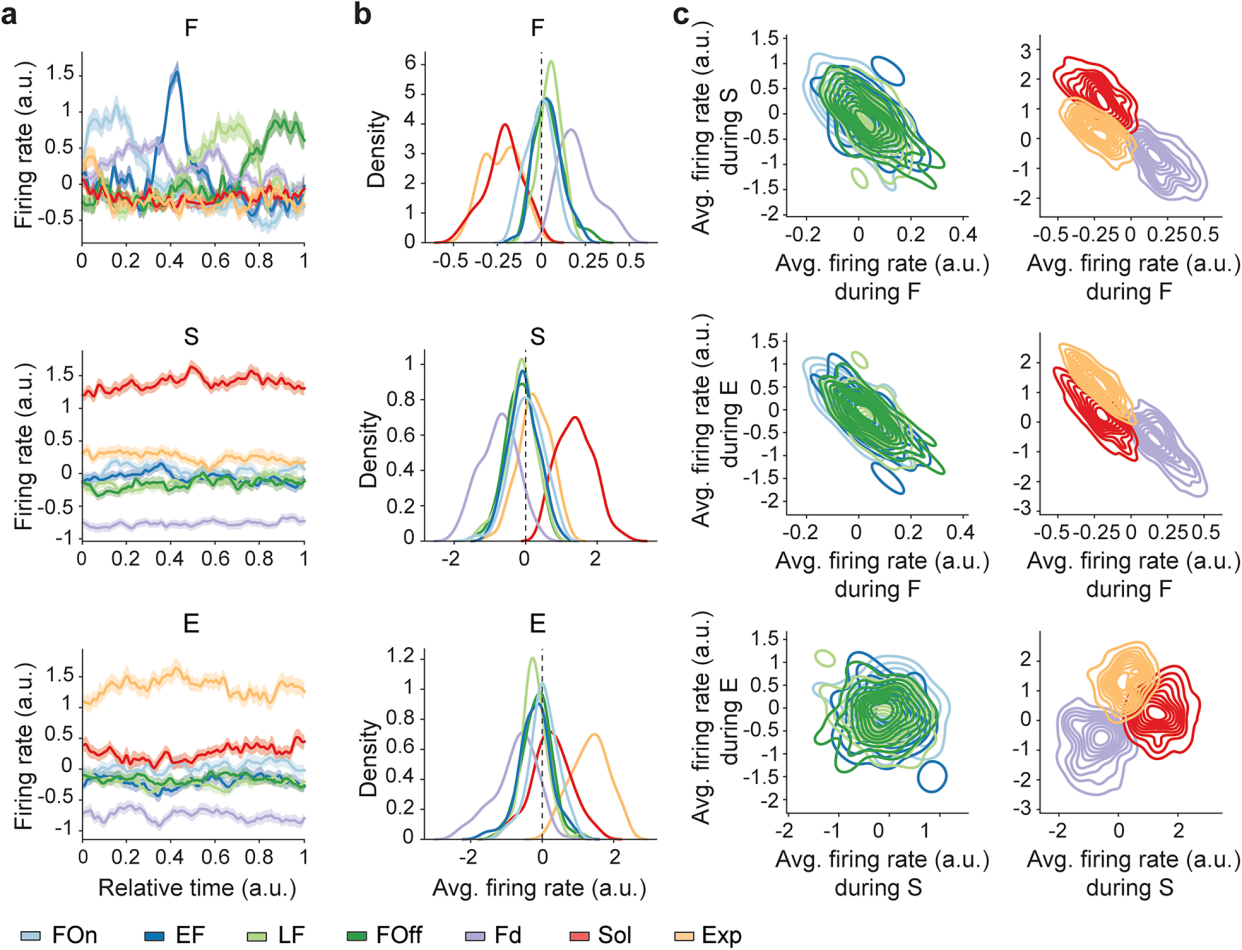
Dynamics and relative activity of functional LH populations during innate behaviors. ***a***, The firing rate of LH populations during feeding (F), social interaction (S), and exploration (E) (a.u., *z*-scored during the three innate behaviors, mean ± SEM across cells). The color code of neuronal populations is maintained throughout the figure. ***b***, Distributions of the average firing rate (kernel density estimation) during behaviors. One-way ANOVA; mean firing rate during feeding, *F*_(6,542) _= 267.81; during social interaction, *F*_6,542 _= 207.46; during exploration, *F*_6,542 _= 137.00; *p* < 0.0001 for all the behaviors. Dashed line, mean of *z*-scored firing rate distributions. ***c***, Correlation of the mean firing rate (a.u., *z*-scored) between the three behaviors for feeding phase (left plots) and other behavior-specific populations (right plots). Contours represent the probability density estimated for each population. Pearson’s correlation between F and S: FOn, *r* = −0.63; EF, *r* = −0.49; LF, *r* = −0.57; FOff, *r* = −0.72; Fd, *r* = −0.56; Sol, *r* = −0.65; Exp, *r* = −0.69; between F and E: FOn; *r* = −0.66; EF, *r* = −0.74; LF, *r* = −0.61; FOff, *r* = −0.78; Fd, *r* = −0.79; Sol, *r* = −0.80; Exp, *r* = −0.84; *p* < 0.0001 for all *r*; between S and E: LF, *r* = −0.31; *p* = 0.0155; other correlations, *p* > 0.05.

Differences in the timing of neuronal activity during feeding and across behaviors evident in individual cells (Figs. 2*a*, 3) were further elucidated by examining the average firing rate dynamics. During feeding, cells from the feeding populations showed a consistently high level of activity, signaling the relative time within feeding episodes (Fig. 4*a*,*b*, Fig. 2*b*). In contrast, during exploration and social interaction, cells from all populations showed temporally homogenous activity, with cells from the exploration and social interaction populations firing at the highest firing rates during these behaviors (Fig. 4*a*,*b*). We next examined the activity of individual LH cells from each population during different behaviors. Activity during feeding was strongly inversely correlated with firing during exploration and social interaction for all populations (Pearson's correlation during feeding vs social interaction; −0.72 ≤ *r* ≤ −0.49; *p* < 0.0001; and vs exploration, −0.84 ≤ *r* ≤ −0.61; *p* < 0.0001; Fig. 4*c*). However, firing during social interaction did not typically predict the response during exploration, suggesting an independent representation of these behaviors in LH (Fig. 4*c*).

**Figure 5.**
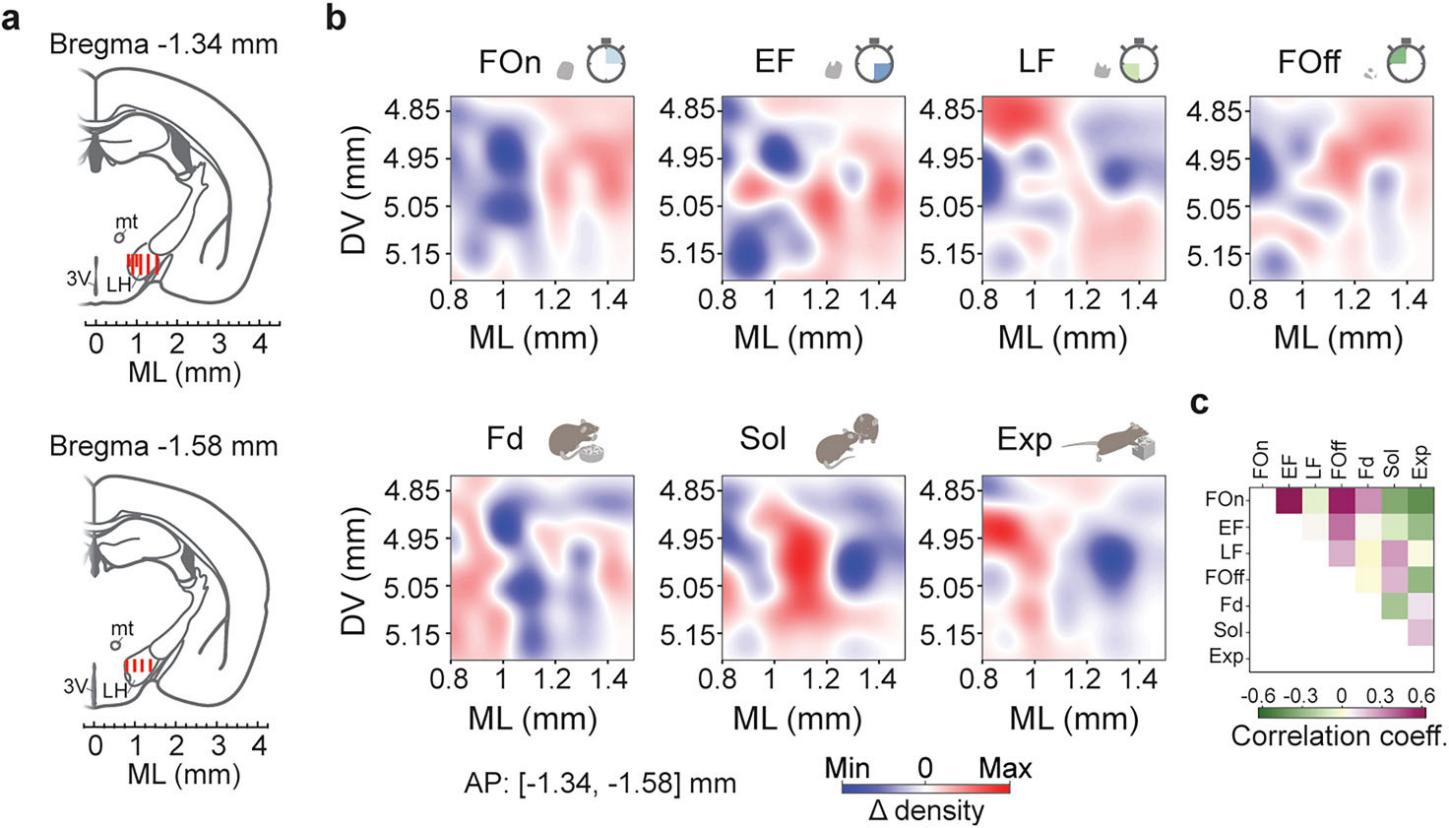
Anatomical localization of functional LH populations. ***a***, Reconstruction of recording electrodes positions in the LH; red lines indicate the path of electrodes’ advancement from the implantation until the last recording session in three mice. ***b***, The difference of the probability density between individual LH populations and all recorded cells at each anatomical location. mt, mammillothalamic tract; 3V, third ventricle. ***c***, Pearson's correlation between histograms shown in ***b***. All correlations are significant, *p* < 0.0422 to *p* < 0.0001, except for EF versus Sol, Fd, LF versus FOn, EF, Fd, Exp, Fd versus FOff, Exp, Sol versus Exp, *p* > 0.05. The correlations were computed on 2D anatomical locations parcellated into 38.8 μm DV bins and 70 μm ML bins, 10 × 10 grid.

Next, we examined the anatomical localization of neurons from the recorded populations within the LH. For this analysis, 505 cells recorded from three mice in close anteroposterior planes (1.34–1.58 posterior to the bregma, Fig. 5*a*) were used. We computed two-dimensional probability density distributions of the recorded cells in mediolateral versus dorsoventral aspects, i.e., according to the shanks of a silicon probe and to their advancement, respectively. The probability density distributions were computed for the individual LH populations and for all the populations combined, the difference between the two probability densities at each location indicates the location-specific enrichment with cells from an individual population (Fig. 5*b*). FOn, EF, and FOff populations featured correlated anatomical distributions; they were enriched in the lateral aspects of LH without clear dorsoventral differences (Fig. 5*b*,*c*). In contrast, the localization pattern of LF cells active during LF differed from FOn, EF, Fd, and Exp (*r* close to zero) and moderately correlated with FOff and Sol (*r* = 0.22, 0.27, respectively). LF cells were enriched in the dorsomedial and ventrolateral LH (Fig. 5*b*). The localizations of Fd, Sol, and Exp, homogenously active during individual innate behaviors, were distinct (Fd and Sol, *r* = −0.25; *p* = 0.0122; Fd and Exp, *r* = 0.11; *p* = 0.30; Sol and Exp, *r* = 0.18; *p* = 0.07) and poorly correlated with the localization of cells active during phases of feeding bouts.

Feeding phase populations of LH neurons had also distinct electrophysiological properties. On the one hand, cells from feeding populations active in the second half of feeding bouts had lower average firing rates during the whole recording than the FOn and EF cells active at the beginning of a feeding bout or cells from the populations homogeneously active during behaviors (1.7 Hz, LF, 1.9 Hz, FOff cells vs 3.2 Hz, FOn, 2.6–3.5 Hz in other populations; *p* < 0.05 in individual comparisons by Mann–Whitney test; Table 1). On the other hand, spike width of LF cells was longer than that of EF and of individual populations of homogeneously active cells (*p* < 0.05 in individual comparisons by Mann–Whitney test; spike width was estimated at 25% of the spike amplitude; Csicsvari et al., 1999).

**Table 1.** Physiological properties of functional LH populations

	FOn	EF	lF	FOff	Fd	Sol	Exp
Aver. rate (Hz)	3.2 ± 0.4	2.6 ± 0.2	1.7 ± 0.1	1.9 ± 0.2	2.7 ± 0.2	2.6 ± 0.2	3.5 ± 0.3
Spike width (ms)	0.36 ± 0.02	0.34 ± 0.01	0.41 ± 0.02	0.37 ± 0.01	0.35 ± 0.01	0.36 ± 0.01	0.35 ± 0.02
Isol. dist. (au)	67 ± 5	59 ± 5	51 ± 3	62 ± 5	59 ± 4	68 ± 4	64 ± 3

Average firing rate, spike width and isolation distance, mean ± SEM across cells in a population (columns). Individual Mann–Whitney tests of average firing rates, *α* = 0.05, FOn versus LF, *U* = 1,660; *p* = 0.0270; FOff, *U* = 1,332; *p* = 0.0203; EF versus LF, *U* = 1,145; *p* = 0.0156; FOff, *U* = 1,400; *p* = 0.0260; Exp, *U* = 1,335; *p* = 0.0148; LF versus Fd, *U* = 1,997; *p* = 0.0099; Sol, *U* = 1,403; *p* = 0.0055; Exp, *p* < 0.0001; FOff versus Fd, *U* = 2,400; *p* = 0.0117; Sol, *U* = 1,757; *p* = 0.0151; Exp, *U* = 965; *p* < 0.0001; Fd versus Exp, *U* = 2,299; *p* = 0.0065; Sol versus Exp, *U* = 1,639; *p* = 0.0050. Individual Mann–Whitney tests of spike widths, *α* = 0.05: EF versus LF, *U* = 1,147; *p* = 0.0161; LF versus Fd, *U* = 2,076; *p* = 0.0225; Exp, *U* = 1,183; *p* = 0.0279.

Together, the populations active in the first half and later but not before the termination of feeding bouts may be enriched in GABA and orexin cells, respectively (Mileykovskiy et al., 2005; Karnani et al., 2013), the two anatomically intermingled LH cell types (Karnani et al., 2013). In particular, the localization of LF cells at the analyzed anteroposterior level dorsally and laterally to fornix and physiological properties of LF match those of orexin cells (Mileykovskiy et al., 2005; Karnani et al., 2013; Diaz et al., 2023). Lateral LH aspects enriched with the Sol population (Fig. 5) feature melanin-concentrating hormone (MCH) neurons involved in social behaviors (Allen Brain Atlas, 2011; Yao et al., 2012; Sanathara et al., 2021; Barretto-de-Souza et al., 2023).

### Joint firing of LH cells during gamma oscillations and nonrhythmic epochs

**Figure 6.**
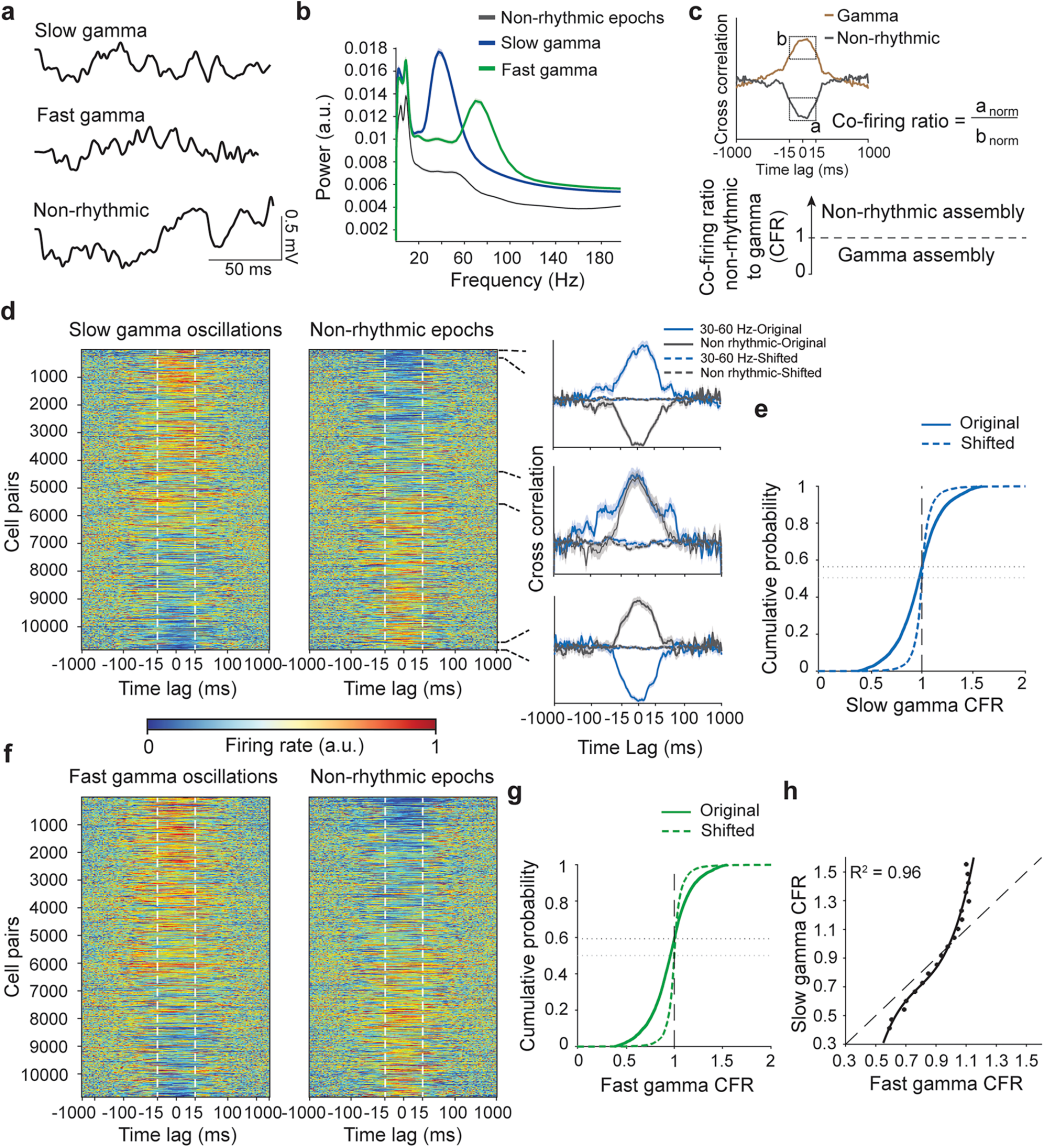
Cofiring of the LH cells during gamma oscillations and nonrhythmic epochs. ***a***, Representative traces of LH LFP gamma oscillations and nonrhythmic epochs (1–100 Hz band pass). ***b***, LFP power spectra during gamma oscillations and nonrhythmic epochs, mean ± SEM across 123 recording sessions from four mice. ***c***, CFR computation and interpretation: nonrhythmic and gamma assemblies. ***d***, Left, CCGs during slow gamma oscillations and nonrhythmic epochs (sorted in the ascending order according to CFR, computed in the window indicated by dashed lines; *n* = 10,838 cell pairs from 4 mice). Right, average cofiring of cell pairs during gamma oscillations and nonrhythmic epochs in the lower and upper 2.5% tails (top and bottom) and middle (20%) of the distribution for original and shifted spike trains. Paired *t* test, CFR original versus shifted, tails, *p* < 0.0001; lower tail, *t*_(257) _= −46.67; upper tail, *t*_(257) _= 62.30; middle, *t*_(2,323) _= −1.57; *p* = 0.12. *X* scale, log. ***e***, Empirical cumulative distribution of slow gamma CFR using original and shifted spike trains (*n* = 10,332 cell pairs from 4 mice). Vertical dashed line, equal cofiring during gamma and nonrhythmic epochs. Horizontal dashed line, fraction of cell pairs with increased cofiring during slow gamma in original (back) and shifted (gray) data. Two-sample KS test, *D* = 0.26; *p* < 0.0001. ***f***, CCGs as in ***d***, for fast gamma oscillations. ***g***, Empirical cumulative distribution as in ***e***, for fast gamma oscillations (*n* = 10,327 cell pairs from 4 mice). Two-sample KS test, *D* = 0.30; *p* < 0.0001. ***h***, Slow and fast gamma CFR in individual cell pairs, averaged in bins of fast gamma CFR and fitted using third degree polynomial (*R*^2^ = 0.96). Dashed line, equal CFR for slow and fast gamma oscillations.

Optogenetic studies suggest that populations of hypothalamic neurons strongly influence innate behaviors when activated synchronously (Jennings et al., 2013; Nieh et al., 2016; Carus-Cadavieco et al., 2017; Kohl et al., 2018). During spontaneous behavior, such collective activity is postulated to require sufficiently strong mutual and/or afferent connectivity of behavior-specific cells to enable their temporal coordination (Hebb, 1949). The formation of cell assemblies can be facilitated by gamma oscillations, as suggested by studies in the hippocampus (K. D. Harris et al., 2003; O'Neill et al., 2008). To investigate assemblies of cells from the functional LH populations, we first characterized joint discharge in the whole population of recorded LH neurons during gamma oscillations and epochs without network oscillations, hereafter nonrhythmic epochs (Fig. 6*a*,*b*). We computed CCGs of 10,838 pairs of LH neurons recorded by different silicon probe shanks, using spikes fired during locally recorded gamma oscillations or nonrhythmic epochs as trigger events. Preferential cofiring during nonrhythmic as compared with gamma oscillation epochs was then quantified as the CFR during these epochs for each cell pair (Fig. 6*c*). The majority of cell pairs increased cofiring during gamma oscillations compared with that during nonrhythmic epochs as indicated by CFR < 1 (Fig. 6*d*–*g*). This agrees with the overall increase in synchronization during gamma oscillations predicted by computational models (Börgers et al., 2008) and demonstrated in the hippocampus (Csicsvari et al., 2003) and in the lateral septum, the main forebrain input of the LH (Risold and Swanson, 1996; Carus-Cadavieco et al., 2017). At the same time, a remarkably high proportion (∼40%) of LH neurons reduced their cofiring to varying degrees compared with nonrhythmic epochs (Fig. 6*d*–*g*). Slow and fast gamma oscillations were associated with different cofiring changes in individual cell pairs. Specifically, cell pairs with low CFR during slow gamma oscillations (i.e., they were more coordinated during slow gamma) showed higher CFR during fast gamma oscillations (i.e., lower coordination during fast gamma; Fig. 6*h*, data points below the diagonal). Conversely, many cell pairs with high CFR during slow gamma had lower CFR during fast gamma (Fig. 6*h*, data points above the diagonal).

### Slow gamma oscillations selectively bind LH cells from feeding-related LH populations

**Figure 7.**
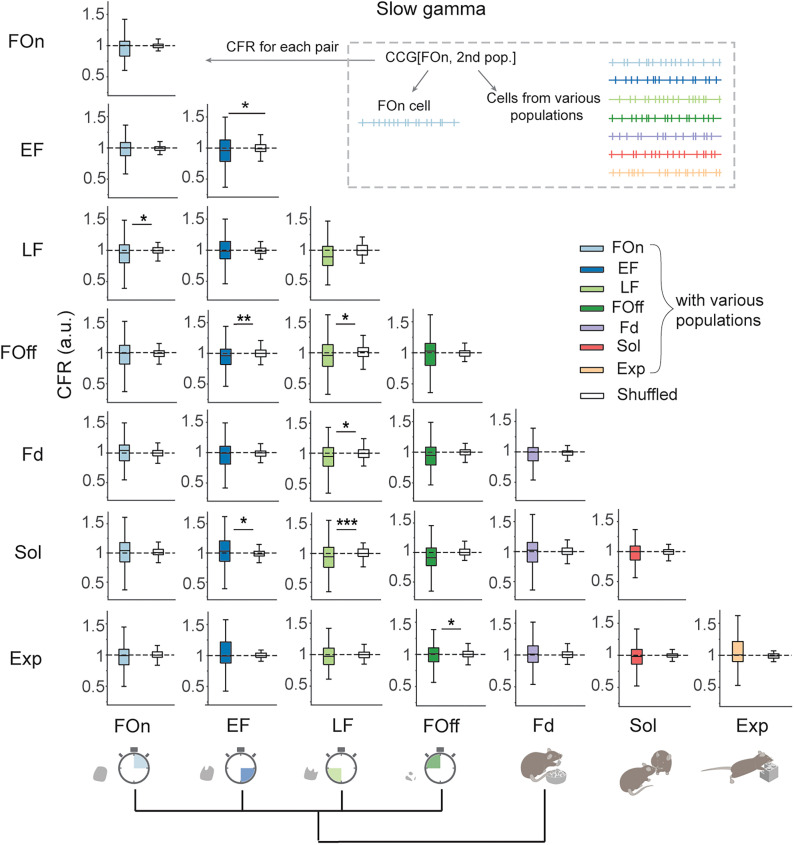
Cofiring of LH cells from functional populations during slow gamma oscillations relative to nonrhythmic epochs. Inset (top right), Scheme of the combinatorial CFR analysis across functional LH populations. Ethological tree diagram with box plots showing CFR of the cells from different functional populations during slow gamma oscillation relative to nonrhythmic epochs in original (colored boxes) and shifted spike trains (empty boxes). Labels of columns and rows, trigger and response populations in CCGs, respectively. Paired *t* test, CFR for original versus shifted spike trains, *α* = 0.05; EF-EF CCG, *t*_(138) _= 2.02; *p* = 0.0458; LF-Fd CCG, *t*_(101) _= 2.57; *p* = 0.0117. Wilcoxon signed-rank test, *α* = 0.05; FOn-LF CCG, *z* = 2.00; *p* = 0.0453; EF-FOff CCG, *z* = 2.78; *p* = 0.0055; LF-FOff CCG, *z* = 2.08; *p* = 0.0379; EF-Sol CCG, *z* = 2.01; *p* = 0.0448; LF-Sol CCG, *z* = 3.34; *p* = 0.0008; FOff-Exp CCG, *z* = 2.13; *p* = 0.0329. Other comparisons, *p* > *α*. Dashed line at CFR = 1, equal cofiring during gamma and nonrhythmic epochs. *p < 0.05, **p < 0.01, ***p < 0.001, ****p < 0.0001.

**Figure 8.**
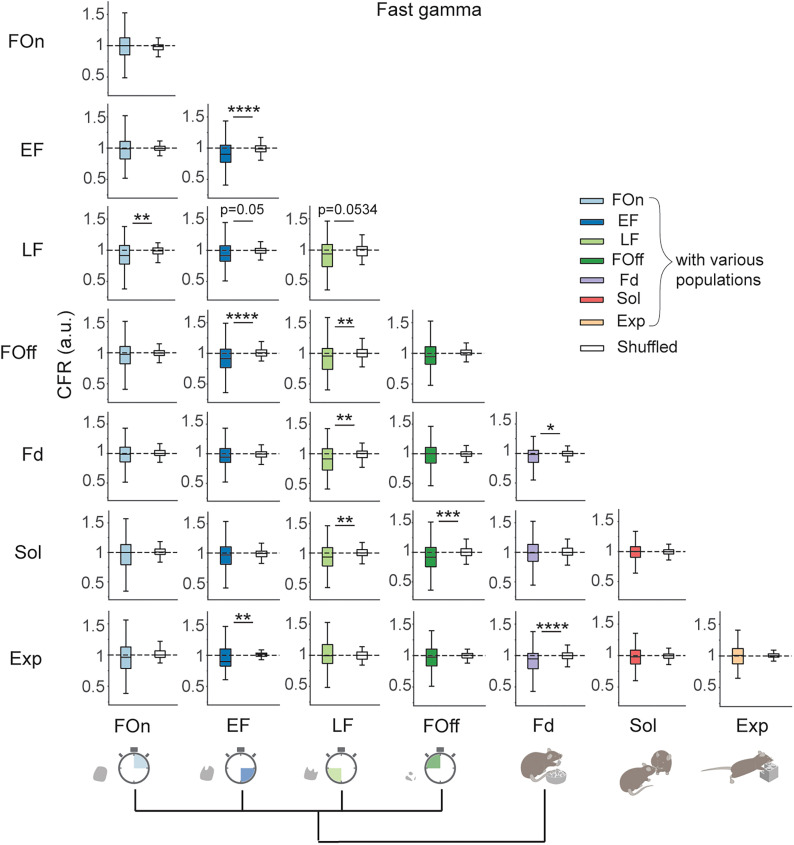
Cofiring of LH cells from functional populations during fast gamma oscillations relative to nonrhythmic epochs. Cofiring of the cells from different functional LH populations during fast gamma oscillations (60–90 Hz) relative to nonrhythmic epochs in original (colored boxes) and shifted spike trains (white boxes). Paired *t* test, CFR for original versus shifted spike trains, *α* = 0.05; EF-LF CCG, *t*_(87) _= 1.99; *p* = 0.0500; LF-LF CCG, *t*_(33) _= 2.00; *p* = 0.0534. Wilcoxon signed-rank test, *α* = 0.05; EF-EF CCG, *z* = 3.89; *p* < 0.0001; EF-FOff CCG, *z* = 4.27; *p* < 0.0001; Fd-Fd CCG, *z* = 2.57; *p* = 0.0101; Fd-Exp CCG, *z* = 4.40; *p* < 0.0001; FOn-LF CCG, *z* = 2.81; *p* = 0.0050; EF-Exp CCG, *z* = 2.82; *p* = 0.0048; LF-FOff CCG, *z* = 2.64; *p* = 0.0084; LF-Fd CCG, *z* = 2.88; *p* = 0.0039; LF-Sol CCG, *z* = 2.71; *p* = 0.0067; FOff-Sol CCG, *z* = 3.36; *p* = 0.0007. Other comparisons, *p* > *α*. Dashed line at CFR = 1, equal cofiring during gamma and nonrhythmic epochs. *p < 0.05, **p < 0.01, ***p < 0.001, ****p < 0.0001.

Finally, we investigated whether the increase in cofiring during gamma oscillations relative to nonrhythmic epochs depends on the behavioral identity of the neurons. To this end, we compared the CFR computed for CCGs of cells from the same or different functional LH populations according to their possible behavioral hierarchy (Tinbergen, 1951) using original and shifted spike trains (Fig. 7). Cells from feeding-related populations, but not from populations preferentially active during different behaviors, were more coordinated during slow gamma oscillations than during nonrhythmic epochs (Fig. 7). Accordingly, the coordination of cells across some of the behavioral populations (EF and Sol, FOff and Exp) was even diminished during slow gamma oscillations. A notable exception was an increased coordination of LF and Sol cells during both slow and fast gamma oscillations (Fig. 8). In contrast, during fast gamma oscillations, the coordination between multiple neuronal populations was increased. This included not only a more prominent cofiring of cell across and within feeding-related populations during fast gamma but also a higher cofiring of neurons from the populations active during feeding, social interaction, and novel object exploration (Sol and LF, Sol and FOff, Exp and EF, Exp and Fd, Fig. 8). Concordantly with uncorrelated firing rates of individual cells between social and exploratory behaviors (Fig. 4*c*), the cofiring of neurons from within Sol and Exp populations was not different during slow or fast gamma oscillations and nonrhythmic epochs. Thus, a high degree of specialization of LH populations in feeding control is accompanied by a distinct network synchronization regime of slow gamma oscillations that preferentially facilitates interactions of LH neurons within and across feeding-related populations.

## Discussion

In this study, using unsupervised spectral clustering of firing rate dynamics, we identified specific LH populations that are sequentially or homogenously active within feeding bouts, as well as populations that are preferentially active during other innate behaviors. This functional parcellation of LH population activity is a behavioral description of its assumption-free segmentation. We show a differential role of gamma oscillations in the coordination of neurons from these emergent populations: slow gamma oscillations by and large synchronize feeding-related neurons, suggesting a novel mechanism for the long-term regulation of food intake, whereas fast gamma oscillations facilitate the coordination of neurons involved in a broader range of behaviors, highlighting their role in the LH function of regulating multiple innate behaviors.

Previous studies have demonstrated an elaborate involvement of neurochemically distinct LH populations in food intake. A population of vesicular GABA transporter (VGAT)-positive LH cells increases activity during feeding and drives food intake (Jennings et al., 2015; Barbano et al., 2016; Nieh et al., 2016; Carus-Cadavieco et al., 2017; C. Chen et al., 2024). Another population also increases its activity during feeding but responds specifically to incentive salient cues during appetitive behaviors and changes its responses in conditioning paradigms (Berridge and Valenstein, 1991; Siemian et al., 2021b; Petzold et al., 2023). This population is composed of cells expressing both VGAT and leptin receptors (Sharpe et al., 2017; Siemian et al., 2021b) as well as a smaller group of cells expressing vesicular glutamate transporter 2 (VGLUT2; Rossi et al., 2021). In the rostrocaudal intermediate part of the LH, where we performed recordings, glutamatergic neurons, which project to the VTA, respond more to aversive (bitter) than to appetitive tastants and do not significantly affect food intake (Jennings et al., 2013; Nieh et al., 2016; Rossi et al., 2021). Some of these cells colocalize orexin, the application of which in the VTA induces burst firing of dopaminergic neurons and regulates reward-seeking and hedonic feeding (Korotkova et al., 2003; G. C. Harris et al., 2005).

Previous electrophysiological recordings in rodents and monkeys have highlighted several types of LH neuronal activity changes in relation to feeding. Firstly, most of the cells in the LH, which is innervated by the gustatory division of the nucleus of the tractus solitarius, change their firing rate in response to various tastants (Norgren, 1970; Burton et al., 1976; Fukuda et al., 1986; de Araujo et al., 2006; J. X. Li et al., 2013) and according to the tastant palatability (J. X. Li et al., 2013). These firing rate changes were closely correlated with the delivery of a tastant or food followed by consumption, typically lasting up to 5 s (Burton et al., 1976; Mora et al., 1976; Fukuda et al., 1986; de Araujo et al., 2006; J. X. Li et al., 2013), which is shorter than spontaneous feeding episodes in our study with a minimal duration of ∼5 s and an average feeding bout of 25–30 s in different analyses. Elevations of the firing rate in response to palatable tastants (lab chow in our study) subside within the first second (J. X. Li et al., 2013), making these neurons possible members of the FOn population active at the very beginning of feeding bouts. The encoding of satiety states (without differences between anticipatory and consummatory phases, de Araujo et al., 2006) and the firing according to phases of learning tasks (Fukuda et al., 1986; Nieh et al., 2015) have also been reported at the abovementioned short timescale and may therefore contribute to the dynamics of FOn rather than other populations active later during self-paced feeding bouts. Secondly, the responses to glucose or other types of food were reported mostly in food- (or water-) deprived or restricted subjects (Burton et al., 1976; Sasaki et al., 1984; Fukuda et al., 1986; Ono et al., 1986; Mileykovskiy et al., 2005; de Araujo et al., 2006; J. X. Li et al., 2013). This makes these mostly feeding-inhibited cells, which have been proposed to be involved in responses to food reward (Rolls et al., 1980; Sasaki et al., 1984), unlikely sole contributors to the LH populations described here in subjects fed *ad libitum*. Under the conditions of the present study (familiarity of food and context, feeding *ad libitum*), food intake is regulated by LH VGAT cells (Siemian et al., 2021a), which could be enriched in the EF populations (FOn and EF) with relatively high average firing rates and short action potentials. In contrast, LF cells active later during feeding bouts exhibit electrophysiological features characteristic of orexin cells and show an anatomical enrichment that correlates with the localization of orexin cells (Mileykovskiy et al., 2005; Karnani et al., 2013; Diaz et al., 2023). Thirdly, distinct LH Sol and Exp populations active during spontaneous nonfeeding behaviors contrast with the paucity of LH neurons active upon the presentation of nonfood objects, interactions with which were not self-initiated (Fukuda et al., 1986). Together, the demonstrated here entire spectrum of firing dynamics within and across naturalistic innate behaviors controlled by the LH is compatible with previously reported early responses during feeding and reveals further sequentially active LH populations with distinct properties. These functionally and, in part, physiologically and anatomically distinct neuronal populations provide the basis for processing different sets of afferents involved in the control of various innate behaviors by the LH (Burdakov and Karnani, 2020).

During feeding bouts, LH is influenced by changing intrinsic and extrinsic inputs. AgRP cells in the arcuate nucleus, which are active before the onset of feeding and are critical for homeostatic hunger, rapidly decrease their activity upon first sensory contact with food (Krashes et al., 2011; Betley et al., 2015; Y. Chen et al., 2015; Sternson and Eiselt, 2017). Food intake is thought to be driven by LH cells that provide efferents to reward- and feeding-related neurons in the VTA and brainstem (Nieh et al., 2016; Rossi et al., 2021). The excitability of these VGAT-positive LH cells is increased during food intake due to a pause in the firing of their afferent nucleus accumbens D1 receptor expressing cells (Roitman et al., 2005; O'Connor et al., 2015). The activity of this specific population of nucleus accumbens neurons is stimulated by VTA cells, which in turn are stimulated by VGAT-positive LH cells. The timing of these neural interactions during eating remains to be determined: they may occur in an alternating sequence at different phases of eating, such as placing food in the mouth, chewing, and swallowing. Over a longer meal timescale, the LH receives various anorectic signals, including gastric distention and intestinal peptide actions on vagal afferents initially integrated in the nucleus of the solitary tract, rising blood glucose concentrations, and falling ghrelin levels acting directly in the LH or conveyed to the LH, along with food palatability signals from the hindbrain (Grill and Hayes, 2012; Ly et al., 2023). Thus, the duration of feeding bouts, and hence the amount of food consumed, likely results from an interplay of bodily feedback anorectic signals and those of appetitive salience, all integrated by LH neurons that drive food consumption. Appetitive drive at the onset of feeding is increased, at least in part, by the activity of MCH neurons (Subramanian et al., 2023). Importantly, the FOff population can either drive feeding close to the end of the episode or terminate it. The latter is probably not the case, as the only LH neurons identified so far that clearly suppress feeding—VGLUT2-positive cells projecting to the lateral habenula—were more abundant in more anterior LH areas than we recorded from in this study (Rossi et al., 2021). This suggests that the LF population can be enriched in the VTA-projecting orexin and possibly other VGLUT2-positive cells. Separate populations of LH feeding phase cells may indicate a specialization within the LH feeding circuitry to process different levels of metabolic and neural anorectic signals at different times of feeding episodes to ensure proper sensory evaluation of food and consumption of its sufficient amount. The involvement of the same classes of feeding time cells in both long and short feeding episodes suggests a potential hypothalamic mechanism for computing the duration of feeding according to metabolic demands, sensory properties of food, and cognitive state.

Neuronal populations active during different phases of a behavioral episode were identified here only for food intake, highlighting a crucial role of LH in the regulation of feeding (Margules and Olds, 1962; Stuber and Wise, 2016). In contrast, LH cells with preferential activity during other innate behaviors showed homogeneous responses during behavioral episodes. Single episodes of social interaction and exploration of novel objects were substantially shorter than feeding bouts. In contrast to feeding, which involves the alternation of basic appetitive patterns and consummatory acts, social interaction and exploration of novel objects are almost entirely appetitive (Tinbergen, 1951) and thus not associated with consummatory negative feedback signals, such as those present during fighting or mating. The feedback signals during these latter behaviors might act similarly to anorectic signals and reduce the activity of the respective LH cell groups. Recordings of social behaviors from lower levels of the ethological hierarchy, including simple consummatory acts such as mating and fighting (Tinbergen, 1951), are needed to investigate possible sequential activity of LH cells during other behaviors.

The hierarchical organization of neural mechanisms that drive many instinctive behaviors is often viewed as hard-wired (Tinbergen, 1951), as opposed to complex behavioral programs shaped by synaptic plasticity that defines experience-specific cell assemblies in the cerebral cortex (Hebb, 1949). However, several lines of evidence point to plasticity in hypothalamic behavioral control circuits, including the conditioning of LH cell responses during pavlovian associative learning with food reinforcement, long-term potentiation of synaptic responses, and enhanced neural responses in the ventromedial hypothalamic aggression locus following repeated agonistic experiences (Stagkourakis et al., 2020) as well as social and sexual experience-dependent segregation of neural representations of the sexes in this hypothalamic area (Remedios et al., 2017). A joint activity of neurons—a prerequisite for sufficiently strong responses of efferent cells and hence for behavioral control—is enabled by shared inputs and mutual interconnectivity and thus relies on spontaneous activity of cells that strengthen these connectivity properties. Building associations with new cells requires repeated coactivation to establish or strengthen synaptic connections. Joint firing is prominent during episodes of increased network excitability, which often coincide with fast network oscillations. Cell assemblies associated with gamma oscillations are easily influenced by inputs, can readily incorporate new neurons (Börgers et al., 2008), and represent episodic memories in the medial temporal lobe of rodents and humans (K. D. Harris et al., 2003; Lopes-Dos-Santos et al., 2018; Roux et al., 2022; Umbach et al., 2022). Gamma oscillations also synchronize LH with the lateral septum and the medial prefrontal cortex, support food seeking (Carus-Cadavieco et al., 2017), and are functionally distinct from LH beta oscillations (15–30 Hz), which provide a temporal framework for encoding behavioral transitions and upcoming behaviors (C. Chen et al., 2024). The present results indicate that slow gamma oscillations differ from fast gamma in enhancing interactions within and between LH populations involved in lower hierarchical levels of feeding, which include elementary appetitive manipulations with food and consummatory acts. Taken together, our results provide behavioral label-free evidence for motivational subsystems in the LH which potentially underlie appetitive behaviors and consummatory acts. Dysfunction of these motivational mechanisms at the basis of behavioral hierarchies may be critical in the pathophysiology of anorexia nervosa, binge eating, and other psychiatric disorders.
